# The Use of Clozapine in Incarcerated Persons with Borderline Personality Disorder

**DOI:** 10.1192/j.eurpsy.2025.1512

**Published:** 2025-08-26

**Authors:** N. Divac, S. Čekerinac, D. Sekulić

**Affiliations:** 1 Dept. of Pharmacology, Clinical Pharmacology and Toxicology, University of Belgrade, Faculty of Medicine, Belgrade; 2 Dept. of Psychiatry, General Hospital of Sremska Mitrovica, Sremska Mitrovica, Serbia

## Abstract

**Introduction:**

Borderline personality disorder (BPD) is common in incarcerated persons. Psychiatric medications are prescribed in prisons for the treatment of psychiatric illnesses, but also for the reductions of symptomes triggered by the specific conditions and environment. The use of psychotropic medications in incarcerated persons is beneficial in terms of prevention of aggression and violent outbursts. Clozapine, as the most effective antipsychotic for aggressive and violent behavior could be very useful in forensic population, but is avoided due to adverse effects and the need for regular monitoring (Cekerinac et al. IJOPH 2024).

**Objectives:**

The objective of this reserch is to analyze the use of clozapine among incarcerated persons with BPD, and to evaluate the incidence of adverse effects.

**Methods:**

A cross-sectional, epidemiological survey was used to measure the prevalence of antipsychotic prescribing among adult prisoners in Sremska Mitrovica Prison (Serbia) in 2020.

**Results:**

Of 1280 incarerated persons, (all men, average age 36.3 years), 80 (6.25%) were prescribed an antipsychotic. More than a half (N=44) were prescribed clozapine, but in doses lower than recommended for approved indications. None of them had an approved indication for clozapine, so this can be defined as off-label use. The average dose of clozapine was 51.14 mg/day, while the recommended maintenance dose is 300–450 mg/day. The other commonly used antipsychotic in this population was olanzapine (N=30). No cases of elevated white blood cells count were noted during regular monitoring. For the broader purpose of the study, metabolic parameters were assessed for the users of both antipsychotics, BMI, plasma glucose levels, plasma cholesterol levels and plasma triglyceride levels. Only the mean values of the levels of glucose and triglycerides in the plasma were slghtly elevated compared to the referent values of the Prison Hospital (Table 1.).

Table 1. Metabolic parameters in inmates prescribed olanzapine and clozapine vs. inmates who were prescribed metabolically inert antipsychotics

**Table:** 

Metabolic parameter (mean±SD)	Treated with clozapine or olanzapine	Referent values
BMI	26.147±4.180	25.0-29.9 (healthy weight)
Glycemia mmol/L	5.783±0.849	3.9-5.6
Cholesterol mmol/L	4.413±0.953	<5.17
Triglycerides mmol/L	2.433±1.380	<1.7

**Conclusions:**

We justify the off-label use of clozapine in prison settings due its benefits in reducing violence and aggression; however, further research would be necessary to clarify does the use of clozapine in incarcerated persons cause behavioral improvements that could reduce recidivism and improve post-imprisonment outcomes. The prevalence of adverse effects is rare; however, that is possibly due to low doses of the prescribed antipsychotics and specific prison settings in terms of dietary options and physical activity, as well as the average young age of the inmates.

**Disclosure of Interest:**

None Declared

